# Comprehensive Analysis and Summary of the Value of Immunophenotypes of Mature NK Cell Tumors for Differential Diagnosis, Treatment, and Prognosis

**DOI:** 10.3389/fimmu.2022.918487

**Published:** 2022-06-24

**Authors:** Qiyao Pu, Xueyan Cao, Yuke Liu, Dongyao Yan, Ran Tan, Jiwei Li, Baohong Yue

**Affiliations:** ^1^ Department of Laboratory Medicine, The First Affiliated Hospital of Zhengzhou University, Zhengzhou, China; ^2^ Key Clinical Laboratory of Henan Province, Zhengzhou University, Zhengzhou, China; ^3^ Department of Oncology, The First Affiliated Hospital of Zhengzhou University, Zhengzhou, China; ^4^ Faculty of Laboratory Medicine, Zhengzhou University, Zhengzhou, China

**Keywords:** mature NK cell tumors, reactive NK cell hyperplasia, FCM immunophenotype, diagnosis and treatment, prognosis

## Abstract

**Background:**

Few studies have been performed to comprehensively analyze and summarize the immunophenotype and differential diagnosis of mature NK cell tumors, and there is often overlap between tumorigenic and reactive NK cell phenotypes. Furthermore, the impact of different phenotypes on patient prognosis has rarely been reported.

**Methods:**

The degree of expression of extracellular and intracellular markers of NK cells in each group was compared by FCM, and the differences in expression of various markers among different disease groups and their impact on prognosis have been analyzed and summarized.

**Results:**

Compared with normal NK cells, tumor cells of ANKL and ENKTL had characteristics of being more activated and progressive with larger FSC, in contrast to NK-CLPD and RNKL. Differential diagnoses with RNKL, ANKL, and ENKTL have broader FCM clues. In contrast, the phenotypes of NK-CLPD and RNKL are not significantly different, and consistent phenotypic abnormalities require ongoing monitoring to confirm malignant clones. The sensitivity of differentiating malignant NK cells from reactive NK cells by KIRs alone was poor. The clustering results showed that CD5, CD16, CD56, CD57, CD94, CD45RA, CD45RO, HLA-DR, KIRs, Granzyme B, Perforin and Ki-67 were differentially distributed in the expression of three NK cell tumors and reactive NK cell hyperplasia, so a comprehensive judgment using a wide range of antibody combinations is required in disease staging diagnosis. The tumor cell loads in BM and PB were also compared, and there was a clear correlation between the two. Moreover, the sensitivity of PB for monitoring tumor cells was up to 87.10%, suggesting that PB could be used as an alternative to BM for the diagnosis and screening of NK cell tumors. Analysis of the phenotypic impact of ENKTL patients on prognosis showed that those with CD7 and CD45RO expression had a poor prognosis, while those with positive KIRs had a better prognosis.

**Conclusion:**

This study systematically characterized the FCM of mature NK cell tumors, emphasizing the importance and clinical value of accurate immunophenotyping in diagnosing, classifying, determining prognosis, and guiding treatment of the disease.

## 1 Introduction

Depending on the stage of NK cell development, they can be divided into tumors of precursor NK cell origin, which are acute leukemias of the undetermined spectrum and have not been widely recognized, and mature NK cell-derived tumors (1). In the current WHO classification, mature NK cell tumors are classified into three disease states, in order, aggressive NK cell leukemia (ANKL), extranodal NK/T lymphoma, nasal type (ENKTL-N), and NK cell chronic lymphoproliferative disorder (NK-CLPD) (2). The course of ANKL is highly aggressive, progresses rapidly, and has a very poor prognosis, with patients often dying within days to weeks of onset. It often involves peripheral blood, bone marrow, liver, and spleen (3). ENKTL is a non-Hodgkin’s lymphoma, characterized by an aggressive clinical course with the nasal and extra-nasal onset and a locally aggressive tumor (4–7). NK-CLPD is a rare malignant lymphoproliferative disease originating from a mature NK cell lineage, with a chronic inert course in most patients without any symptoms and usually without EBV infection (8, 9).

In clinical practice, certain immune stimuli can lead to an overlap between the immune phenotype of reactive NK cells and that of neoplastic NK cells. Distinguishing RNKL with an altered immunophenotype from tumorigenic NK cells, especially chronic NK cell proliferative disease (CLPD-NK), is extremely challenging (10, 11). To explore the specific clinical value of FCM analysis in the diagnosis and management of mature NK cell tumors and reactive NK cell hyperplasia, in this study, we retrospectively analyzed and summarized the immunophenotypic features of a large number of NK cell tumors.

For exploring the status of NK cells holistically, FCM is an analytical tool with great advantages in precisely detecting indicators of clonality, activation status, proliferation status, and degree of depletion of NK cell tumors. We found that performing multiple iterations of FCM assessment to confirm phenotypic concordance abnormalities facilitated the differentiation between tumorigenic NK cells and reactive NK cells. Furthermore, we demonstrate that FCM immunophenotyping is an important tool for the diagnosis of tumorigenic NK cells and is valuable for the classification and treatment of NK cell tumors, indicating prognosis and selection of potential therapeutic targets.

## 2 Materials and Methods

### 2.1 Patients and Controls

Bone marrow or peripheral blood samples were collected from patients with aggressive NK cell leukemia (ANKL, n=14), extranodal NKT lymphoma invading bone marrow (ENKTL, n=23), chronic NK cell proliferative disease (NK-CLPD, n=8), and reactive NK lymphocytosis (RNKL, n=10) admitted to the Department of Oncology of the First Affiliated Hospital of Zhengzhou University from March 2019 to April 2022. Anemia patients with non-NK cell tumor-related diseases during the same period in our hospital were screened as normal bone marrow NK cells specimens (NNK, n=20) controls and other hematopoietic cells showed no abnormal morphology and phenotype.

Among them, 44 patients with mature NK cell tumors and 10 patients with reactive NK cells had complete clinical data. The diagnosis and classification of ANKL, ENKTL, and NK-CLPD were based on the 2016 World Health Organization (WHO) diagnostic criteria. Demographic information, clinical data, laboratory results, and examination images were collected from our electronic medical records. All subjects have given written informed consent to the Declaration of Helsinki. This project was approved by the Ethics Committee of the First Affiliated Hospital of Zhengzhou University [IRB ID: 2021-KY-1132-002].

### 2.2 Bone Marrow Smear or Blood Smear, Laboratory Test Results of a Routine Blood Test, Blood Biochemical Test, and EBV-DNA Copy Number Test

Bone marrow fluid was aspirated by a puncture. Bone marrow fluid or peripheral blood was smeared, dried, and stained with Wright-Giemsa staining. Since there are many nucleated cells in the bone marrow fluid, the staining time of the smear is 10 minutes, and the staining time of the peripheral blood smear is 7 minutes.

The absolute numbers of white blood cells, red blood cells, hemoglobin, and platelets were measured using a fully automatic hemacytometer Beckmann COULTER LH750. LDH, ALP, β 2-Mg, and ferritin in serum were detected by Roche Cobas C 701 spectrophotometric automatic analyzer. PCR was used to detect the DNA level of the EB virus in the blood.

### 2.3 Erythrocytes in Bone Marrow or Peripheral Blood and Preservation of Cell Suspension After Lysis

#### 2.3.1 Materials

50mL tip bottom tube, 15mL tip bottom tube, 12×75 Falcon tube, slide, disposable pipette, ribbon, and 110µm nylon mesh. Bone marrow fluid containing K_2_-EDTA anticoagulant, peripheral blood containing anticoagulant K_2_-EDTA.

CPM cell preservation solution: 10% fetal or newborn calf serum and various antibiotic cell preservation media (CPM) are added to RPMI or Hanks buffer to maintain the viability of lymphocyte suspension prepared in the laboratory. CPM can normally maintain high cell viability for several days. In addition, the medium can be used as a diluent to regulate the concentration of cell suspension.

#### 2.3.2 Lysis of Red Blood Cells in Bone Marrow

Place a glass slide on the tip of a 50mL centrifuge tube, and the bone marrow fluid flows slowly into the centrifuge tube along with the glass slide (the glass slide can be used to stick off part of the grease and observe whether there is a clot in the bone marrow fluid and treat it in time). Add a sufficient amount of erythrocyte lysate in the ratio of bone marrow fluid: lysate =1:10. If the sample is severely agglutinated, grind it.

#### 2.3.3 Lysis of Red Blood Cells in Peripheral Blood

The peripheral blood samples were placed in a 50mL pointy bottom centrifuge tube, and 3 ~ 4 drops of blood were collected in the original specimen tube. No more than 2.5-3mL of peripheral blood should be dripped into the 50mL pointy-bottom centrifuge tube, and the ratio of blood: lysate =1:15 should be added into the RBC lysate, but no more than 25mL. If necessary, another pointy-bottom centrifuge tube should be taken. If the sample is severely agglutinated, grind it.

After the lysis process, the cells were restored in CPM solution. The cell suspension was filtered with 110μm nylon mesh into another labeled 50mL tip bottom centrifuge tube. Cell count in 50mL tip bottom centrifuge tube was recorded. Total number of cells obtained = volume of cells in 50mL tip bottom centrifuge tube × white blood cell count measured by the instrument (total number of cells was recorded on the print sheet); Adjust the concentration of cell suspension in each tube to 3.0×10^6^/mL.

### 2.4 Flow Cytometry Sample Preparation and Data Analysis

Add an appropriate amount of cells to each tube (according to the total number of cells, cell viability, etc.), and the number of cells should not exceed 1×10^6^. Add the corresponding antibody combination to the 5 reagent tubes respectively ①CD57-FITC/CD8-PE/CD3-PerCP/CD56-PECy7/CD4-APCCy7/CD7-V450/CD45-V500/PD-1-BV605, ②CD3-FITC/CD2 -PE/CD5-PerCP/CD56-PECy7/CD4-APCCy7/CD7-V450/CD45-V500, ③Ki-67-FITC/CD30-PE/CD3-PerCP/CD56-PECy7/HLA-DR-APC/CD7-V450/CD45-V500/CD38-BV605, ④CD158e1-FITC/CD158b-PE/CD3-PerCP/CD56-PECy7/CD158i-AF647/CD16-APCCy7/CD158a-BV421/CD45-V500,⑤Granzyme B-PE/CD3-PerCP/CD56-PECy7/CD94-APC/CD16-APCCy7/Perforin-BV421/CD45-V500, among them, tube 4 and tube 5 need to do cell membrane breaking treatment. See **Supplementary Table 1** for the clone number of the antibodies used. Shake and ice bath in the dark for 15 minutes. Add 2 mL of CPM to each tube, centrifuge at 500g for 5 minutes, and aspirate the supernatant. Repeat the wash twice. Add 300 μL of sheath fluid to resuspend cells. Store in the dark at 4°C and put on the machine within 2 to 4 hours. After exclusion of duplex cells and debris, lymphocytes were gated using lateral scattering (SSC) with CD45 dot plots. NK cells were gated according to CD56 expression and CD3 deletion, NK cells were defined as CD3-CD56+ events, and CD16+ and CD7+ were applied to correctly identify NK cells in cases where CD56 was lost. The expression of immune markers of NK cells was then further analyzed. (Data obtained using BD Fcs Diva and Fcs Canto II). FCS data were analyzed and interpreted using Kaluza software.

### 2.5 Statistical Methods and Analysis

The categorical data in the Tables are tested by Fisher’s Precision probability method. The mean value of normally distributed variables is tested by an unpaired T-test. The Kruskal-Wallis nonparametric test was used to analyze median differences in non-Gaussian data. Pearson’s and Spearman’s were used to evaluating the positive and negative correlation between measurement variables. The expression of immune markers in different patient populations was analyzed by hierarchical clustering, and the correlation among different markers was analyzed. Kaplan-Meier survival analysis was used for cumulative survival. P < 0.05 was considered statistically significant (*p < 0.05, **p < 0.01, ***p < 0.001, ****p < 0.0001). Data were processed and analyzed using GraphPad Prism 9.0 (San Diego, CA, USA), SPSS Version 26.0 (SPSS, Chicago, IL), and R4.0.3 (R Foundation, Vienna, Austria).

## 3 Result

### 3.1 Clinical Features of Patients With Mature NK Cell Tumors

The demographic and clinical characteristics of the included cases are shown in **Table 1**. Three disease groups were prone to multiple lymphadenopathies, splenomegaly, hyperlipidemia, hypoalbuminemia, thrombocytopenia, increased RDW, increased ALP, and increased β2-MG during the onset of the disease, and there was no significant difference in symptoms. Compared with the ENKTL and NK-CLPD patient groups, the ANKL group had higher rates of fever and hepatomegaly. Hemophagocytic syndrome and low hemoglobin are more common in ANKL. Compared with patients in the ANKL group and ENKTL group, the median age of onset of NK-CLPD patients was older, about 54 years. Anemia, cytopenia, abnormal coagulation function, abnormal LDH level, and elevated ferritin were lower in NK-CLPD. At the same time, ANKL patients are more prone to kidney damage and physical fatigue caused by hemophagocytic factor storms in the blood than ENKTL patients. The proportion of liver function damage in the NK-CLPD group was significantly lower than that in the ENKTL group. Compared to NK-CLPD groups, there are more EBV viral loads in the blood of ANKL groups. Reactive NK cell hyperplasia is less likely to present with these symptoms. **Supplementary Table 2** demonstrates the p-values indicating the differences between the groups.

**Table 1:** Clinical characteristics of cases.

	No. (n/N) (%)				
	ANKL (N=14)	ENKTL (N=23)	NK-CLPD (N=8)	RNKL (N=10)	NNK (N=20)
Age, years, mean (SD)	34.31 (14.65)	38.43 (11.80)	54.29 (3.90)	51.40 (5.83)	47.70 (3.92)
Male sex	8 (57.14)	17 (73.91)	4 (50.00)	4 (40.00)	8 (40.00)
**Symptoms at onset of illness**					
Fever	14 (100.00)	16 (69.57)	5 (62.50)	3 (30.00)	1 (5.00)
Fatigue	12 (85.71)	8 (34.78)	5 (62.50)	3 (30.00)	5 (25.00)
Multiple lymph nodes are swollen throughout the body	7 (50.00)	15 (65.22)	2 (25.00)	0	0
Hepatomegaly	8 (57.14)	2 (8.70)	1 (12.50)	0	0
Splenomegaly	12 (85.71)	17 (73.91)	4 (50.00)	2 (20.00)	3 (15.00)
**Complications**					
Hemophagocytic syndrome	12 (85.71)	9 (39.13)	2 (25.00)	1 (10.00)	0
Hyperlipidemia	10 (71.43)	13 (56.52)	4 (50.00)	1 (10.00)	2 (10.00)
Hypoproteinemia	8 (57.14)	8 (34.78)	1 (12.50)	0	3 (15.00)
Hepatic dysfunction	14 (100.00)	20 (86.96)	4 (50.00)	1 (10.00)	2 (10.00)
Renal dysfunction	9 (64.29)	5 (21.74)	2 (25.00)	0	2 (10.00)
Coagulation dysfunction	13 (92.86)	16 (69.57)	3 (37.50)	2 (20.00)	5 (25.00)
Multiple organ failure	7 (50.00)	5 (21.74)	0	0	0
**Laboratory results**					
White blood cell <4 × 10^9^/L	7 (50.00)	15 (65.22)	3 (37.50)	3 (30.00)	7 (35.00)
Neutrophil <2 × 10^9^/L	5 (35.71)	7 (30.43)	4 (50.00)	2 (20.00)	5 (25.00)
Red blood cells<4 × 10^9^/L	14 (100.00)	19 (82.61)	3 (37.50)	2 (20.00)	14 (70.00)
Hemoglobin<105 g/L	13 (92.86)	13 (56.52)	4 (50.00)	3 (30.00)	11 (55.00)
Platelets<100 × 10^9^/L	11 (78.57)	18 (78.26)	3 (37.50)	3 (30.00)	14 (70.00)
RDW> 15%	11 (78.57)	16 (69.57)	5 (62.50)	2 (20.00)	13 (65.00)
Serum LDH > 250 U/L	12 (85.71)	17 (73.91)	2 (25.00)	4 (40.00)	4 (20.00)
Serum ALP > 100 U/L	12 (85.71)	14 (60.87)	3 (37.50)	2 (20.00)	5 (25.00)
Serum β2-MG > 3 mg/L	11 (78.57)	16 (69.57)	3 (37.50)	3 (30.00)	1 (5.00)
Serum ferritin > 150 ng/mL	14 (100.00)	22 (95.65)	1 (12.50)	2 (20.00)	2 (10.00)
Serum EBV-DNA >5.0 × 10^3^ copies/mL	13 (92.86)	15 (65.22)	3 (37.50)	1 (10.00)	0

ANKL, Aggressive NK-cell leukemia; ENKTL, Extranodal NK/T-cell lymphoma; NK-CLPD, Chronic NK cell lymphoproliferative disease; RNKL, Reactive NK lymphocytosis; NNK, Normal NK cells; RDW, Red blood cell distribution width; LDH, lactate dehydrogenase; ALP, Alkaline phosphatase; β2-MG, β2 microglobulin.

### 3.2 Summary of Immunophenotyping of ANKL, ENKTL, NK-CLPD, and RNKL Based on Multicolor Flow Cytometry

**Figure 1:**
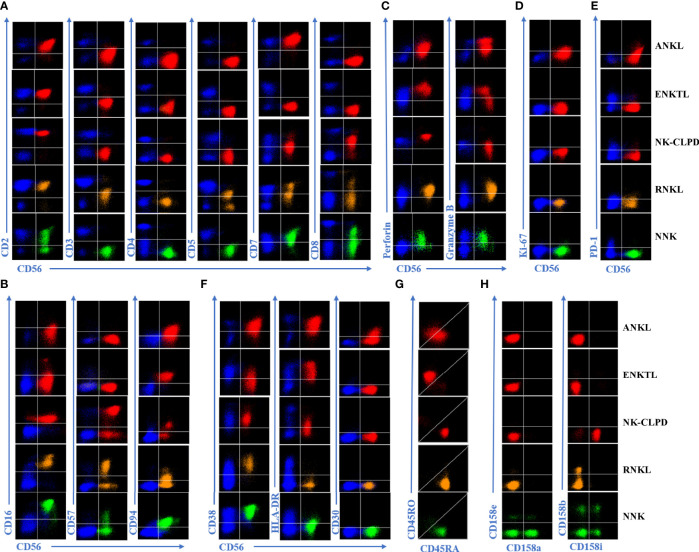
Expression of different extracellular and intracellular markers in ANKL, ENKTL, NK-CLPD, RNKL, and normal NK cells. **(A)** Cell surface pan-lineage-associated markers were analyzed by flow cytometry. Representative FACS plots show the expression of CD2, sCD3, CD4, CD5, CD7, and CD8 in tumorigenic NK cells, reactive NK cells, and normal NK cells. **(B)** Analysis of lineage-specific markers of NK cells. Representative FACS graphs show the expression of CD16, CD56, CD57, and CD94 in each group of NK cells. **(C)** Analysis of functional markers of NK cells. Graphs show the expression of Granzyme B as well as Perforin in each group of NK cells. **(D)** Representative FACS graph showing the expression of Ki-67, a proliferation marker for each group of NK cells. **(E)** Representative FACS graphs showing the expression of the depletion marker PD-1 in each group of NK cells. **(F)** Representative FACS graphs showing the expression of activation markers CD30, CD38, and HLA-DR in each group of NK cells. **(G)** Analysis of the differences between CD45RA and CD45RO in aberrant NK cells. Representative FACS graphs show the expression of abnormal indicator markers CD45RA and CD45RO in each group of NK cells. **(H)** The Clonality of NK cells was determined by analyzing the expression distribution of KIRs. Representative FACS graphs show the expression of CD158a, CD158b, CD158e, and CD158i in each group of NK cells. Red scatter dots represent tumorigenic NK cells, yellow scatter dots represent reactive NK cells, and green scatter dots represent normal NK cells. The blue scatters on the graph represents other normal T lymphocytes in the background and can be used as a negative or positive control for marker expression.

The proportion distribution and phenotype of normal NK cells have a lot in common. FCM immunophenotyping mainly diagnoses abnormal NK cells according to the changes of various numbers and expression markers compared with normal cells. **Figure 1** is an example of the expression of each marker in three groups of mature NK cell tumors, the reactive control group, and the normal control group. **Supplementary Table 1** shows the combination of antibodies.

The phenotypic abnormalities in the four groups of cases with ANKL, ENKTL, NK-CLPD, and RNKL were as follows. (1) Proportion of cases with abnormal expression of NK cell-associated antigens. ANKL, 100%; ENKTL, 100%; NK-CLPD, 100%; RNKL, 100%. (2) The proportion of lymphocytes occupied by NK cells is More than 40%, and the proportion is significantly higher than normal (10%~20%). ANKL, 100%; ENKTL, 69.56%; NK-CLPD, 75%; RNKL, 20%; (3) The proportion of NK cells appear monoclonal, i.e., the proportion of KIRs showing restricted expression. ANKL, 92.86%; ENKTL, 91.30%; NK-CLPD, 87.5%; RNKL, 70%.

Under normal physiological conditions, NK cells can be divided into two subpopulations. They are CD56+briCD16-/dim NK cells, also known as naive NK cells, which produce a large number of cytokines upon stimulation by pro-inflammatory factors, and CD56+dimCD16+ NK cells, known as activated NK cells, which mainly mediate cytotoxic effects and have a strong killing capacity (12–14). We divided NK cells from 20 normal controls into the above two subpopulations to analyze their immunophenotype. It was found that NK cells in the CD56+ bright subpopulation exhibited characteristics of CD2+bri/CD7+bri in addition to the characteristic of not expressing KIRs. Nevertheless, because the proportion of this subset of NK cells was quite low, accounting for only about 5% of all NK cells, it did not affect the result of scattered expression of KIRs when normal NK cells were analyzed as a whole.

We compared the immunophenotypes of the three disease groups and the RNKL group with the two subpopulations of normal NK cells and summarized their characteristics in **Table 2**. Compared with normal NK cells, the main characteristics of ANKL cells are: CD7-/+het, CD16+dim/-, CD56+bri, CD57-, CD94+bri, HLA-DR+, Granzyme B+het/-, Perforin+ het/-, KIRs-, Ki-67+ or PD-1+ or CD30+, abnormal expression of CD45RA and CD45RO. The main features compared to ENKTL are: CD7-/+het, CD8-, CD16-, CD56+bright, CD57-/+het, CD94+/+bri, HLA-DR+, Granzyme B+het/-, Perforin+het/-, KIRs-/+het, KI-67+/dim or PD-1+ or CD30+, abnormal expression of CD45RA and CD45RO. The main features of NK-CLPD are CD7-/+het, CD16+/-, CD56-/+dim, CD57-/+het, KIRs-/+het, Granzyme B+het/-, Perforin+het/-. The main features of RNKL are CD5+dim/-, CD7+/+dim, and CD16+/+dim. The phenotypes of ANKTL and ENKCL partially overlapped, and both showed more activated phenotypes: HLA-DR+, Ki-67+/dim, CD45RO-/+het.

**Table 2:** FCM immunophenotypes of mature NK cell tumors, reactive NK cells and normal NK cells.

	CD2	CD3	CD4	CD5	CD7	CD8	CD16	CD56	CD57	CD94	CD38	HLA-DR	CD45RA	CD45RO	Perforin	Granzyme B	KIRs	Ki-67	PD-1	CD30
ANKL	+	–	–	–	-/+ het	-/dim	+dim/-	bri	–	bri	+	+/-	+/-	-/+	+het/-	+het/-	–	+/-	-/+	–
ENKTL	+	–	–	–	-/+ het	–	–	bri	-/+ het	bri	+/bri	+/-	+/-	-/+	+het/-	+het/-	-/+ het	+/-	-/+	–
NK-CLPD	+	–	–	–	-/+ het	-/dim	+	+/-	+ het	+	+	+/-	+	–	+het/-	+het/-	-/+ het	–	-/+	–
RNKL	+	–	–	–	+/ dim	-/dim	+	+	+het	+	+	–	+	–	+	+	+het/-	–	–	–
Activated NK cells	+	–	–	–	+	-/dim	+	dim	-/+	dim/-	+	-/dim	+	–	+	+	+	–	–	–
Naive NK cells	bri	–	–	–	bri	-/dim	-/dim	bri	–	bri	+	–	+	–	-/dim	-/dim	–	–	–	–

het, heterogeneous expression; bri, bright expression; Activated NK cells, CD56+dimCD16+ NK cells; Naive NK cells, CD56+briCD16-/dim NK cells.

**Figure 2:**
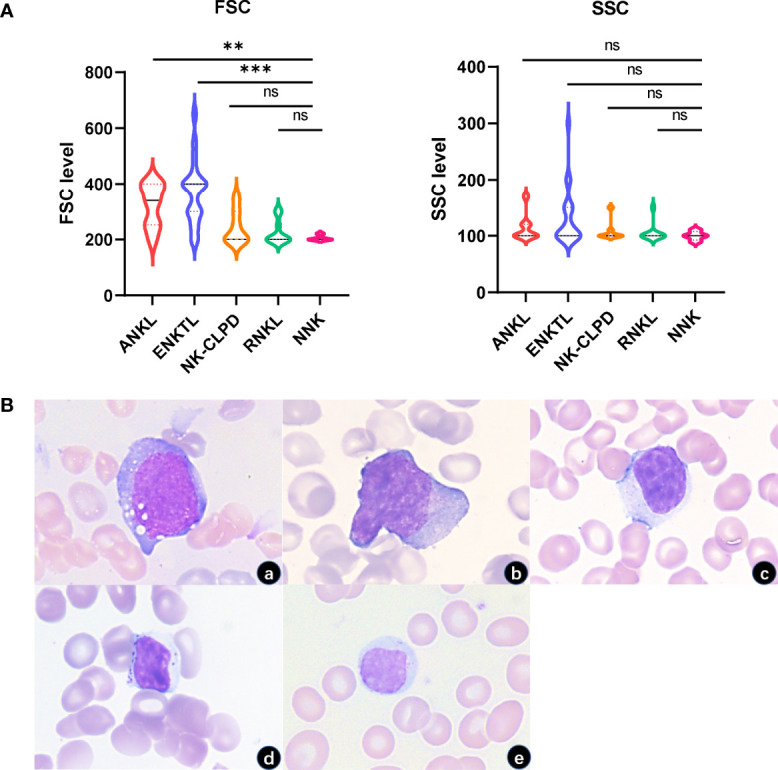
FSC and SSC characteristics of NK cells in each group. **(A)** FSC of tumor cells in ANKL and ENKTL were significantly higher than those of normal NK cells and reactive NK cells. There was no significant difference in SSC between the NK cells of each group. **(B)** Morphologically tumorigenic NK cells in ANKL and ENKTL were significantly larger with or without granules in the cytoplasm (a, ANKL; b, ENKTL; c, NK-CLPD; d, RNKL; e, NNK; 1000×) (**p<0.01, ***p<0.001). ns, no significant difference.

FSC and SSC of 5 groups of cells were analyzed. By comparing FSC, we found that the tumor NK cells in the ANKL and ENKTL groups were generally larger than NK-CLPD tumor cells (P<0.05; P<0.01), and were morphologically verified. However, the volume of NK cells in the NK-CLPD and RNKL groups was not significantly different from normal NK cells. Consistent with morphology, there were no significant differences in SSCs between neoplastic NK cells and normal NK cells **(** **Figure 2**).

### 3.3 Phenotype-Based Cluster Analysis of ANKL, ENKTL, NK-CLPD, and RNKL

**Figure 3:**
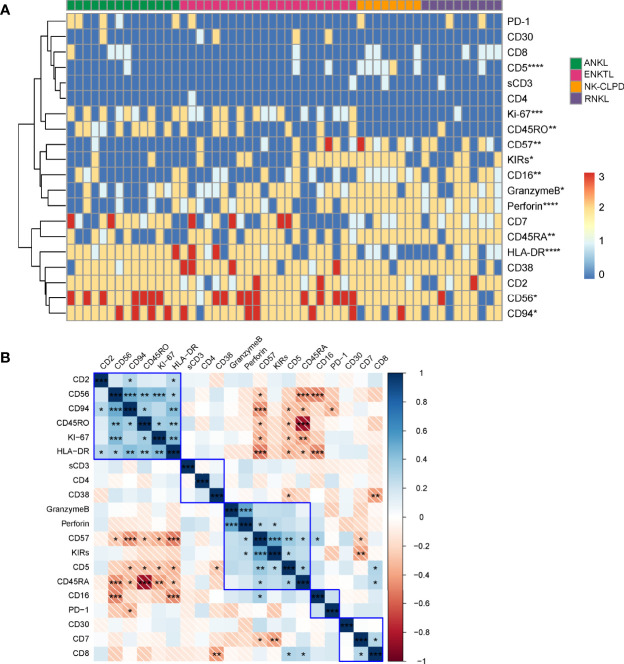
Phenotype-based cluster analysis of ANKL, ENKTL, NK-CLPD and RNKL. **(A)** Clustering of 20 immunomarkers of NK cells from patients with ANKL (n = 14), ENKTL (n = 23), NK-CLPD (n = 8) and RNKL (n = 10) Graph. The horizontal axis is for each individual patient and the vertical axis is for the immune markers. **(B)** Correlation matrix of the 20 immunomarkers. (*p < 0.05, **p < 0.01, ***p < 0.001, ****p < 0.0001).

To evaluate the effectiveness of multicolor flow cytometry in distinguishing between disease groups of mature NK cell tumors, we semi-quantitatively converted 23 immunomarkers of patients into FCM scores (neg=0; +weak=1; +=2; +str=3) according to their expression intensity, and the exact method of defining expression intensity can be seen in **Supplementary Figure 1**. Hierarchical clustering was subsequently performed, and the results are shown in **Figure 3**. The clustering plots clearly show that FCM is very effective in differentiating disease groups. Using the Kruskal-Wallis method to analyze the variability of expression of each marker among different disease groups, we found that CD5, CD16, CD56, CD57, CD94, CD45RA, CD45RO, HLA-DR, KIRs, Granzyme B, Perforin, and Ki-67 were differentially distributed among the four groups. Nevertheless, no significant differences were seen in the expression of CD2, CD3, CD4, CD7, CD8, CD30, CD38, and PD-1 between the disease groups **(** **Figure 3A** **)**. By further correlation analysis of the expression of each marker, we found a positive or negative correlation among the specific markers for the diagnosis of NK cell tumors (**Figure 3B**).

### 3.4 Characteristics of the Reactive NK Cell Hyperplasia

The cases of reactive NK cell hyperplasia (RNKL) collected in this experiment were all discovered incidentally during flow cytometry analysis of bone marrow for other clinical indications. Among them, 1 had a viral infection, 1 had autoimmune disease, 1 had Hodgkin’s lymphoma, 2 had thrombocytopenia, and 5 had diffuse large B-cell lymphoma (One of the cases was EBV + DLBCL). Clinically, reactive NK cell hyperplasia manifests as an increase in the absolute number of NK cells in the peripheral blood, with an increase in large granular lymphocytes seen in bone marrow smears or peripheral blood smears. It is often caused by immune stimulation produced by the primary disease and the clonal NK cells will disappear in a short period of time **(** **Figures 2A–E** **)**. A summary of flow cytometry immunophenotyping of 10 RNKL cases. Abnormal immunophenotypes were found in 6 (60%) cases. The proportions of complete deletion of CD2, CD7, CD16, CD38, CD57, and CD94 were 20% (2/10), 20% (2/10), 10% (1/10), 10% (1/10), 20% (2/10), 30% (3/10). The proportions of abnormal expression of sCD3, CD5, CD8, HLA-DR and PD-1 were 10% (1/10), 60% (6/10), 30% (3/10) and 10% (1/10), respectively. The reduction ratio of CD2, CD7, and CD16 expression intensity was 10% (1/10), 30% (3/10), and 5% (5/10), respectively. Abnormal NK cell function (attenuated or absent expression of Granzyme B or Perforin) was found in 1 (10%) case. Restricted expression of KIRs was found in 9 (90%) cases, of which 30% (3/10) were all negative for KIRs, and 40% (4/10) restricted expression of a subset of KIRs. The researchers followed up patients with RNKL, and the reactive NK cells in the bone marrow of 10 patients disappeared within 3 months to half a year.

### 3.5 Tumor NK Cells and Reactive NK Cells Were Identified by FCM

**Figure 4:**
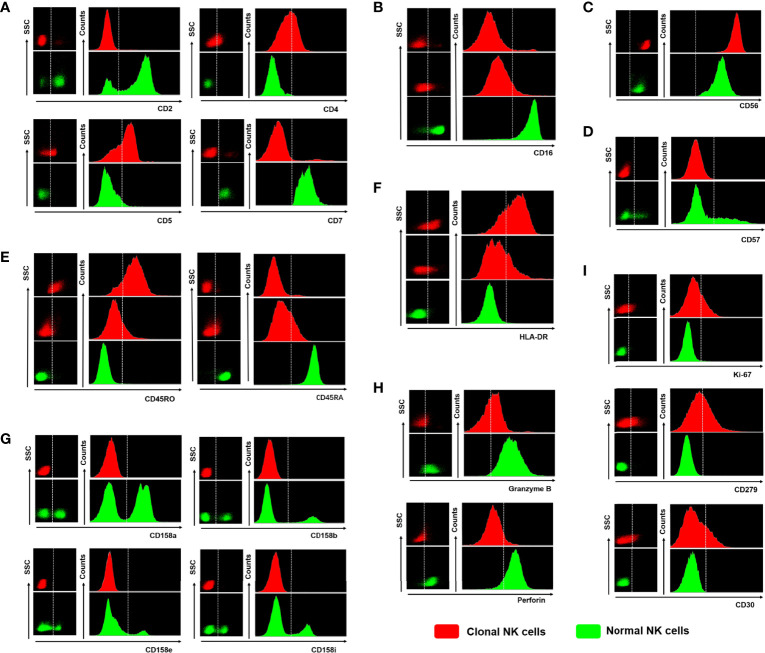
The differences in expression based on the specific markers between clonal NK cells and normal NK cells. **(A)** Abnormal expression or loss of CD2, CD4, CD5, and CD7 (The phenotype of normal NK cells is usually CD2+CD4-CD5-CD7+). **(B)** Homogeneous attenuation or loss of CD16 (normal NK cells CD16+). **(C)** Strong expression of CD56 (stronger than normal NK cell CD56 expression level). **(D)** Loss of CD57 homogeneity (heterogeneous scattered expression of CD57 in normal NK cells). **(E)** CD45RA-/CD45RO+ or CD45RA+dim/CD45RO+dim (normal NK cells express CD45RA+CD45RO-). **(F)** Positive or weakly positive expression of HLA-DR (normal NK cells do not express HLA-DR). **(G)** None KIRs expression (normal NK cells have scattered expression of all subtypes of KIRs). **(H)** Perforin or Granzyme B is not expressed (normal NK cells express Perforin+/Granzyme B+). **(I)** Ki-67 or CD279 (PD-1) or CD30 positive expression (normal NK cells do not express any of the three). Red scattered dots in the graph represent clonal NK cells and green scattered dots represent normal NK cells.

Relative to the phenotype of normal NK cells, **Table 3** summarizes the expression characteristics of some specific markers of tumorigenic NK cells (ANKL, ENKTL, NK-CLPD) and reactive NK cells (RNKL), which were used to identify tumorigenic NK cells and reactive NK cells by counting and analyzing their frequency of occurrence. The differential expression characteristics of these specific markers are shown in **Figure 4**. Altered expression of lineage markers such as CD2, CD4, CD5 and CD**7** are not characteristic features of neoplastic NK cells. These basal lineage markers in reactive NK cells are also frequently aberrantly expressed. Due to the overlapping phenotypes of malignant NK cells, these basic phenotypes alone cannot qualitatively distinguish benign and malignant NK cells. CD16, CD56, CD57, and CD94 are specific markers of NK cells. CD16 in ANKL and ENKTL groups were often lost or attenuated uniformly (p=0.005; p<0.001), and CD57 was also prone to uniform loss (p<0.001; p=0.020), while RNKL was mainly positive. In the RNKL group, there were no cases with strong CD56 expression, while in the ANKL and ENKTL groups, the proportions were relatively high (50.00% and 43.48%, respectively; p=0.019; p=0.015). Compared to ENKTL and NK-CLPD, ANKL is prone to CD45RO positivity or CD45RA and CD45RO co-positivity or co-negativity (p=0.002), so CD45RA and CD45RO can be used as aberrant markers to distinguish ANKL from RNKL. HLA-DR was expressed in most of ANKL and ENKTL, and was lower in RNKL (p<0.001; p=0.006). A subset of patients with ANKL and NKTCL were negative for cytotoxic effector phenotype markers (Granzyme B and Perforin) compared with RNKL, while RNKL was mostly positive for these markers (p<0.001; p=0.021). RNKL generally does not express Ki-67, CD30, and PD-1, which indicate the active proliferation of functional exhaustion, but ANKL and ENKTL are sometimes expressed (p=0.013; p=0.001). In contrast to ANKL and ENKTL, there was no significant difference in the expression of markers between NK-CLPD and RNKL, and it was difficult to distinguish only by immunophenotype.

**Table 3:** Markers to distinguish mature NK cell tumors (ANKL, ENKTL, NK-CLPD) and reactive NK lymphocytosis (RNKL).

	①ANKL N = 14	②ENKTLN = 23	③NK-CLPD N = 8	④RNKLN = 10	P value			
					(①vs.④)		(②vs.④)	(③vs.④)
Abnormal expression or loss of T-series generic markers (CD2、CD4、CD5、CD7)	8 (57.14)**^a^**	15 (65.22)**^b^**	5 (62.50)**^c^**	4 (40.00)**^d^**	0.680	0.257		0.637
CD16 is uniformly lost or attenuated	10 (71.43)	20 (86.96)	2 (25.00)	1 (10.00)	0.005*	<0.001*		0.559
Strong CD56 expression	7 (50.00)	10 (43.48)	1 (12.50)	0	0.019*	0.015*		0.444
CD57 is uniformly lost	13 (92.86)	16 (69.57)	5 (62.50)	2 (20.00)	<0.001*	0.020*		0.145
Abnormal expression of CD45RA and CD45RO	9 (64.29)	7 (30.43)	1 (12.50)	0	0.002*	0.073		0.444
HLA-DR positive or weak positive	14 (100.00)	19 (82.61)	6 (75.00)	3 (30.00)	<0.001*	0.006*		0.153
None KIRs expression	12 (85.71)	13 (56.52)	5 (62.50)	3 (30.00)	0.010*	0.259		0.342
Granzyme B and/or Perforin negative	12 (85.71)	13 (56.52)	2 (25.00)	1 (10.00)	0.001*	0.021*		0.559
Ki-67 or PD-1 or CD30 expression	9 (64.29)	17 (73.91)	3 (37.50)	1 (10.00)	0.013*	0.001*		0.275

**a**, All 8 cases had loss or weak expression of CD7. **b**, CD7 was lost or weakly expressed (11 cases); CD2 was lost (2 cases); CD4 was expressed (1 case); CD4 expression was accompanied by CD7 loss (1 case). **c**, CD7 was lost or weakly expressed (4 cases); CD5 expression with weak CD7 expression (1 case). **d**, CD5 expression with diminished CD7 expression (3 cases); CD7 loss (1 case). *P-value less than or equal to 0.05 was considered statistically significant.

### 3.6 Cloning Analysis of NK Cells

KIRs (CD158 Series) are a strong indicator of NK cell cloning. This study analyzed four anti-cell immunoglobulin sample receptors (including CD158a, CD158b, CD158e1, and CD158i). In the three tumors NK cell groups and RNKL control groups, the expression patterns of KIRs can be divided into three types, KIRs homogeneous negative; A certain subtype positive in KIRs, other subtypes are not expressed; KIRs dispersion expression. Due to the short-term monoclonal amplification of NK cells in the reaction, the clonal proliferation of NK cells can be identified by KIRs. However, KIRs are significantly different from the expression pattern in normal control NK cells and tumor NK cells or reactive NK cells **(** **Figure 1–H** **)**. In each individual, all normal NK cells express these four KIRs and are thought to have a heterogeneous expression pattern. Thus, restricted KIRs expression (homogeneous positive or negative) suggests that NK cell monoclonal proliferation.

### 3.7 Circulating NK Cells Can Be Used for Screening and Diagnosis of NK Cell Tumors

**Figure 5:**
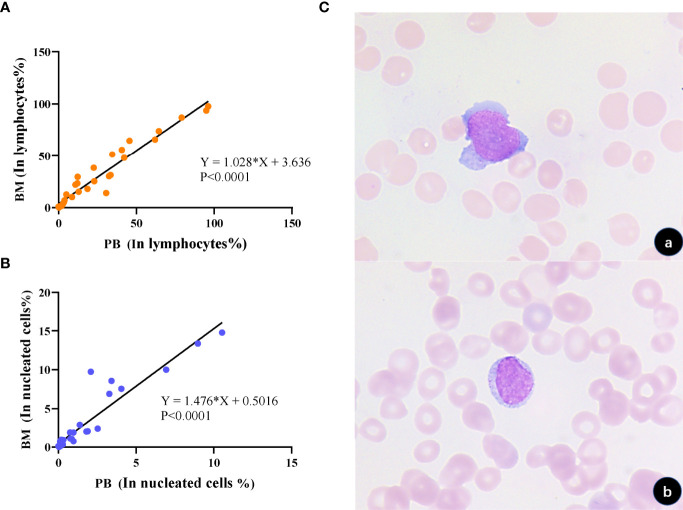
Correlation and homology of tumorigenic NK cell load in the bone marrow and peripheral blood. **(A)** Correlation analysis of the proportion of tumorigenic NK cells occupying lymphocytes in the bone marrow and peripheral blood, respectively, showed a positive correlation (p < 0.0001). **(B)** Correlation analysis of the proportion of tumor NK cells occupying nucleated cells in the bone marrow and peripheral blood, respectively, showed a positive correlation (p < 0.0001). **(C)** Morphological analysis verified that tumor cells in the bone marrow and peripheral blood had similar morphology (a, BM; b, PB; 1000×).

Flow cytometry analysis on peripheral blood was performed during the same period (48 hours) in 31 patients with tumor NK cells detected in bone marrow at the initial diagnosis or after treatment. Among them, circulating tumor cells were found in the peripheral blood of 27 patients, and the detection rate was as high as 87.10%. The analysis of abnormal cells in peripheral blood by flow cytometry and morphology confirmed that they have the same phenotype and morphology as abnormal NK cells in bone marrow, which can prove that they are homologous cells. The proportion of abnormal circulating NK cells (CNKC) and abnormal bone marrow NK cells in lymphocytes and nucleated cells were positively correlated (R=1.028, P <0.001; R=1.476, P <0.001) **(** **Figure 5** **)**. According to the regression fitting line, when tumor cells occupy 3.64% of lymphocytes or less than 0.50% of nucleated cells in bone marrow, tumor cells in peripheral blood may not be detected by FCM. The specificity of detecting NK tumor cells in peripheral blood was 100%, and the sensitivity was 87.10%. This suggests that circulating NK cells could replace disseminated NK cells in the screening and diagnosis of NK cell tumors.

### 3.8 Immunophenotype-Based Prognostic Analysis of ENKTL

**Figure 6:**
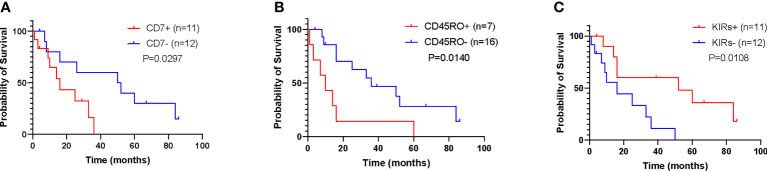
Prediction of survival of ENKTL by immunophenotype (n = 23). **(A)** Effect of CD7 expression on survival time of patients. **(B)** Effect of CD45RO expression on survival time of patients. **(C)** Effect of expression of KIRs on survival time of patients.

Compared to the extremely short survival of ANKL and the long-term survival of NK-CLPD, there was large individual variability in the survival time of ENKTL patients. Their 1-, 3-, and 5-year survival rates were 78.95%, 52.63%, and 36.84%, respectively. Twenty-three ENKTL patients were analyzed, all of whom received sequential treatment with either DDGP (Dexamethasone + Cisplatin + Gemcitabine + Pepmonase) or DDGP + IMRT regimens, with four of them co-administering PD-1 inhibitors late in treatment. To explore the effect of immunophenotype on the survival of ENKTL, we further analyzed the cumulative survival of ENKTL patients and the effect of these markers on survival using the Kaplan-Meier method. We found that the expression of CD7 and CD45RO was negatively correlated with the survival time of patients (p=0.0297, p=0.0140), while the expression of KIRs was positively correlated with the survival time (p=0.0108). Comparing survival time under the influence of each marker at a predicted survival rate of 10%, the expression of CD7 had the greatest impact on survival time and the worst prognosis **(** **Figure 6** **)**. By analyzing univariate Cox proportional risk ratios, it was determined that these three immunomarkers combined with age, PINK score, peripheral blood EBV-DNA, LDH level and β2-MG level jointly affected the survival of ENKTL patients (P < 0.05); Cox multivariate analysis showed that PINK score and EBV-DNA and CD45RO and KIRs expression were prognostic independent clinical factors (P < 0.05) **(** **Table 4** **)**.

**Table 4:** Univariate and multivariate Cox regression analysis affecting the survival time of ENKTL.

Factor	Univariate analysis			Multivariate analysis		
		HR	95% CI	*P*-Value	HR	95% CI	*P*-Value
Sex	(Male/Female)	0.861	0.293-2.532	0.771			
Age	(> 60 years old, ≤ 60 years old)	6.439	0.221-18.600	0.027	NS		
Primary site	(Non nasal, Nasal)	0.616	0.218-1.735	0.165			
PINK Scores	(High risk, Others)	3.422	0.880-13.310	0.022	1.921	0.280-13.199	0.036
B symptoms	(Yes, No)	2.193	0.832-5.779	0.222			
EVB-DNA	(>5.0 × 10^3^ copies/mL, ≤5.0 × 10^3^ copies/mL)	1.791	0.532-6.037	0.008	1.800	0.212-15.257	0.029
LDH	(>245 U/L, ≤245 U/L)	2.824	0.924-8.634	0.006	4.748	1.773-53.589	0.009
β2-MG	(>3 mg/L, ≤3 mg/L)	2.533	0.729-8.809	0.122			
CD7	(Positive, Negative)	2.498	0.894-6.978	0.030	NS		
CD45RO	(Positive, Negative)	3.046	0.900-10.310	0.014	8.729	1.381-30.513	0.021
KIRs	(Positive, Negative)	0.336	0.122-0.927	0.011	0.213	0.025-1.826	0.019

NS, no significance.

## 4 Discussion

NK cell tumors are highly heterogeneous and specific in terms of biological behavior, prognosis, and response to therapy (3, 4, 8). Therefore, a clear understanding of disease typing is particularly important for the subsequent treatment of the disease, which helps clinical grasp of disease progression and precise drug use. Currently, the overall understanding of the immunophenotype of oncogenic NK cells is not well established. This study attempts to elucidate the comprehensive immunophenotypic landscape of NK cells, including T-series universal markers (CD2, sCD3, CD4, CD5, CD7, and CD8), NK lineage-specific markers (CD16, CD56), differentiation markers (CD94, CD57), activation markers (CD30, CD38, HLA-DR), functional markers of cytotoxicity (Granzyme B and Perforin), proliferation markers (Ki-67), depletion markers (PD-1), clonality markers (KIRs: CD158a, CD158b, CD158e1, CD158i), and other markers to determine NK cell abnormalities (CD45RA and CD45RO) (15–17).

Due to the difference in the incidence rate, previous studies have focused more on the immunophenotypic features of ENKTL and often used bone marrow biopsy immunohistochemistry (18, 19). As it often takes 7-10 days from sampling to pathological analysis yet, it makes the diagnosis time-consuming and increases the risk of disease progression. The immunohistochemical single-stain technique does not distinguish well between tumor cells and inflammatory cells, and some other confounding factors may bias the results (20, 21). Fewer studies have performed immunophenotypic analysis of NK tumor cells by flow cytometry. the ENKTL and NK-CLPD phenotypes described by Sanjay de et al. are similar to our cohort study, with ENKTL showing a susceptibility to loss of CD16 and strong expression of CD56, and NK-CLPD being close to the RNKL phenotype (22). However, they did not include cases of ANKL, and the number of immunomarkers analyzed was low and did not sufficiently take into account comprehensive information on the activation and clonality of tumorigenic NK cells (indicators such as CD30, CD38, CD158 series, KI-67, PD-1, CD45RA and CD45RO), which is a limitation.

Compared with these shortcomings, the present study has some advantages. First, FCM can obtain as many target cells as possible, quickly, and perform a high-efficiency analysis of cell size, cytoplasmic granularity, and differentiation antigens. Second, the characteristic immunophenotypic analysis of bone marrow NK tumor cells by FCM can be used as an important indicator for diagnosing the disease, shortening the time to confirm the diagnosis, and facilitating the early diagnosis and rapid clinical management. Third, to describe more information, we simultaneously studied as many extracellular and intracellular immune markers as possible, which resulted from the implementation of a comprehensive immunophenotype monitoring program in our clinical practice. Fourth, the immunophenotypes of the three NK cell tumors and reactive NK cells were comprehensively compared cross-sectionally. The common abnormalities of NK cells based on FCM can be summarized as follows: (1) altered intensity of marker expression, including over-or under-expression and loss of expression (2) expression of other series of antigens (3) presence of markers that are not or rarely expressed by normal NK cells (4) heterogeneous expression of markers that become (5) changes in forward scatter (FSC) and/or side scatter (SSC); (6) monoclonality of mature NK cells.

Unlike malignant clones of NK cells, reactive changes in NK cells are often caused by immune stimuli, such as autoimmune diseases, viral infections, chemotherapy, and stem cell transplantation. A short-term reactive proliferation of NK cells can be triggered, presenting a reactive polyclonal process or a secondary monoclonal process (10, 11). This can be specifically manifested by an altered immune phenotype and/or abnormal expression of KIRs. Their changes disappear after the disappearance of the stimulus. The progression of both is shown in **Supplementary Figure 2**. The overlapping immunophenotypes of reactive and neoplastic NK cells pose a great challenge for the diagnosis of tumors. KIRs are a powerful indicator for detecting NK cell clonality (23, 24). However, our experience shows that reactive NK cells are also frequently detected with restricted expression of KIRs, and thus KIRs are less sensitive for distinguishing between benign and malignant clones of NK cells, requiring the use of a broad antibody panel. We summarized some clues that facilitate the differentiation of ANKL and ENKTL from RNKL by comparing the FCM characteristics of tumor NK cells and reactive NK cells **(** **Table 2** **)**. (1) diminished or lost CD16 homogeneity; (2) strong CD57 expression; (3) CD57 homogeneity loss; (4) HLA-DR expression; (5) Granzyme B and/or Perforin negative; (6) Ki-67 or PD-1 or CD30 expression. Relative to ENKTL and NK-CLPD, abnormal CD45RA/CD45RO expression and all-negative KIRs were more inclined to indicate the occurrence of ANKL. NK-CLPD has too many phenotypic similarities to RNKL to be identified by FCM alone. RNKL tends to disappear within six months, and confirming the consistency of the abnormal expression of each marker by repeating the FCM assay can help distinguish between NK-CLPD and RNKL. The presence of mutations in the STAT3 and STAT5b genes has also been reported to help discriminate between NK-CLPD and reactive NK cell proliferation. And it can be used as a new diagnostic indicator of NK-CLPD disease. However, its frequency is variable, typically occurring in 30% of cases (25–29). Moreover, the immunophenotypic abnormality of NK cell tumors is not an isolated event and must be accompanied by other abnormalities, which can be diagnosed in combination with clinical signs and comprehensive medical findings. We therefore recommend regular follow-up and analysis together with multidisciplinary indicators.

It is clear that the detection of NK cells in peripheral blood has three characteristics compared to bone marrow aspiration testing: the test is less aggressive and not significantly invasive, which improves patient compliance; the absolute CNKC count is more accurate compared to bone marrow NK cells due to the dilution of peripheral blood and the possible focal distribution of tumor cells; CNKC correlates with bone marrow NK cell tumor load in the diagnosis of NK cell tumors. Therefore, compared with the detection of bone marrow NK cells, CNKC detection by FCM has the advantages of high accessibility, applicability, rapidity, and cost-effectiveness, and is the most applicable method despite the lack of sensitivity. Equally as a noninvasive means of monitoring ENKTL disease, circulating EBV-DNA load has been identified as a valid parameter for the diagnosis of ENKTL (7, 30). Circulating EBV-DNA load greater than 5.0 × 10^3^ copies/mL was detected in 65.22% of the patients with primary and recurrent ENKTL included in this study **(** **Table 1** **)**. Nonetheless, the detection rate of peripheral blood tumor NK cells by FCM was above 80%. Therefore, we recommend using FCM to detect circulating NK tumor cells while further obtaining more comprehensive and valid information about the target cells, which can be supplemented by EBV-DNA assay as an adjuvant test.

In summary, we have the following recommendations for the diagnosis of NK cell tumors by FCM. (1) A combined quantitative and qualitative analysis strategy is needed, and a larger and more comprehensive combination of NK cell-related markers is required to improve the sensitivity of the analysis. (2) For the diagnosis of NK cell tumors, experimental diagnostic immunophenotyping to assess clonality in addition to adequate tumor load (32% to 97% of targeted cells) and other abnormal immunophenotypes are required. (3) The interpretation of benign and malignant clonal results should be done with caution and needs to be combined with other examination results for comprehensive judgment. (4) Circulating NK cells in peripheral blood can be detected during disease review to reflect tumor load, efficacy, and disease progression, which can improve patient compliance and thus increase the review rate.

It is brought to our attention that some studies have shown that the expression of CD7 molecules is associated with disease aggressiveness, drug resistance, and poor prognosis (31, 32). Even so, the prognostic significance of other markers for NKTCL has been less reported. To further investigate the impact of different phenotypes on the prognosis of NK cell tumors, this study analyzed the relationship between the expression levels of immune markers and survival time in ENKTL patients. Consistent with the findings of Siding Wei et al, the survival of CD7-positive ENKTL was significantly shorter than that of negative patients (31, 32). Also, we found that patients positive for KIRs had a relatively better prognosis, whereas CD45RO positivity suggested a poor prognosis. However, since the cases in this study were all from a single center, there may be a geographical bias in the sample, and whether these markers can be used as independent reference indicators to determine the prognosis of NKTCL still needs to be validated in large samples and multicenter studies.

Chimeric antigen receptor T cell (CAR-T cell)-mediated cellular immunotherapy is a novel precision-targeted therapy that has been proven to achieve good results in clinical oncology treatment in recent years (33, 34). Laboratory studies on CD7 have shown that CD7 chimeric antigen receptor T cells (CD7 CAR-T cells)-mediated cellular immunotherapy has a great potential to kill CD7-positive hematologic tumor cells (34–36). Therefore, CD7 CAR-T cells may hold greater promise for the treatment of patients with CD7-positive NKTCL. Meanwhile, targeted drugs such as PD-1 inhibitors and PD-L1 inhibitors have been reported to achieve good results in some patients with solid and hematologic tumors (37–41), which have great therapeutic implications for patients with PD-1 or PD-L1 positivity. Since most patients are NK cell CD38 positive, CD38 monoclonal antibodies may become a new direction for NK cell tumor therapy (42). For CD30-positive patients, CD30 monoclonal antibodies may also be used as a means of treatment (43–45). In conclusion, precise immunophenotypic analysis is of great importance for indicating therapeutic targets and developing individualized treatment regimens.

To our knowledge, the present study is the most comprehensive report summarizing the series of NK cell tumor characteristics. We describe in detail the immunophenotypic characteristics and differentiation points of three mature NK cell tumors from reactive NK cells and explain the critical clinical value of FCM in the diagnosis of NK cell tumors, the selection of therapeutic targets, and the determination of prognosis. Still, pathological immunohistochemistry is the “gold standard” for the diagnosis of NK cell malignancies, and flow cytometry can be a useful adjunct. We predict that FCM will play a key role in screening prognostic factors and guiding therapy for NK cell tumors and that an immunophenotype-based prognostic risk scoring system can be further developed.

## Data Availability Statement

The original contributions presented in the study are included in the article/**Supplementary Material**. Further inquiries can be directed to the corresponding author.

## Ethics Statement

The studies involving human participants were reviewed and approved by the Ethics Committee of the First Affiliated Hospital of Zhengzhou University. The patients/participants provided their written informed consent to participate in this study.

## Author Contributions

BY, QP, YL, and JL designed the study. QP, XC, JL, and DY performed experiments. QP, DY, and RT collected the clinical information and classified the patients. QP performed the statistical analysis and drafted. All authors contributed to the article and approved the submitted version.

## Funding

This work was supported by the Natural Science Foundation of Henan Province [grant numbers 162300410299], the Henan Medical science and technology public relations project [grant numbers 2018020007, LHGJ20190038].

## Conflict of Interest

The authors declare that the research was conducted in the absence of any commercial or financial relationships that could be construed as a potential conflict of interest.

## Publisher’s Note

All claims expressed in this article are solely those of the authors and do not necessarily represent those of their affiliated organizations, or those of the publisher, the editors and the reviewers. Any product that may be evaluated in this article, or claim that may be made by its manufacturer, is not guaranteed or endorsed by the publisher.

## Supplementary Material

Supplementary Figure 1 Specific methods for defining expression intensity. According to the amount of expression of surface markers, they can be classified as non-expressed, expressed, weakly expressed, and strongly expressed.

Supplementary Figure 2 Different progression of reactive and malignant NK cells. Reactive NK cells arise in response to immune stimulation and can induce reactive polyclonal or secondary monoclonal proliferation. Reactive NK cells tend to disappear after the disappearance of the stimulus.

Supplementary Table 1 The clone number of the antibodies used.

Supplementary Table 2 p-values for differences between groups.

## Abbreviations

ANKL, aggressive NK cell leukemia; ENKTL-N, extranodal NK/T lymphoma, nasal type; NK-CLPD, NK cell chronic lymphoproliferative disorder; RNKL, reactive NK lymphocytosis; NNK, CNKC, circulating NK cells; normal bone marrow NK cells; BM, Bone Marrow; PB, peripheral blood; FCM,Flow Cytometry; APC, Allophycocyanin; APC-Cy7, Allophycocyanin Cy7; AF647, Alexa Fluor^®^ 647; BD, Becton Dickinson; BV421, Brilliant Violet 421; FITC, Fluorescein isothiocyanate; PE, Phycoerythrin; PECy7, Phycoerythrin Cy7; PerCP, Peridinin chlorophyll protein; V500, Violet 500.

## References

[1] Vardiman JW Thiele J Arber DA Brunning RD Borowitz MJ Porwit A. The 2008 Revision of the World Health Organization (Who) Classification of Myeloid Neoplasms and Acute Leukemia: Rationale and Important Changes. Blood (2009) 114(5):937–51. doi: 10.1182/blood-2009-03-209262 19357394

[2] Cazzola M. Introduction to a Review Series: The 2016 Revision of the Who Classification of Tumors of Hematopoietic and Lymphoid Tissues. Blood (2016) 127(20):2361–4. doi: 10.1182/blood-2016-03-657379 27069255

[3] Ishida F. Aggressive Nk-Cell Leukemia. Front Pediatr (2018) 6:292. doi: 10.3389/fped.2018.00292 30364049 PMC6191480

[4] Yang CF Hsu CY Ho DM. Aggressive Natural Killer (Nk)-Cell Leukaemia and Extranodal Nk/T-Cell Lymphoma Are Two Distinct Diseases That Differ in Their Clinical Presentation and Cytogenetic Findings. Histopathology (2018) 72(6):955–64. doi: 10.1111/his.13463 29314186

[5] Sánchez-Romero C Bologna-Molina R Paes de Almeida O Santos-Silva AR Prado-Ribeiro AC Brandão TB. Extranodal Nk/T Cell Lymphoma, Nasal Type: An Updated Overview. Crit Rev Oncol Hematol (2021) 159:103237. doi: 10.1016/j.critrevonc.2021.103237 33493634

[6] Liu ZL Bi XW Liu PP Lei DX Jiang WQ Xia Y. The Clinical Utility of Circulating Epstein-Barr Virus DNA Concentrations in Nk/T-Cell Lymphoma: A Meta-Analysis. Dis Markers (2018) 2018:1961058. doi: 10.1155/2018/1961058 30581497 PMC6276475

[7] Cho J Kim SJ Park S Yoo KH Ki CS Ko Y. Significance of Circulating Epstein-Barr Virus DNA Monitoring After Remission in Patients With Extranodal Natural Killer T Cell Lymphoma. Ann Hematol (2018) 97(8):1427–36. doi: 10.1007/s00277-018-3313-x 29627879

[8] Zambello R Teramo A Barilà G Gattazzo C Semenzato G. Activating Kirs in Chronic Lymphoproliferative Disorder of Nk Cells: Protection From Viruses and Disease Induction? Front Immunol (2014) 5:72. doi: 10.3389/fimmu.2014.00072 24616720 PMC3935213

[9] Barilà G Calabretto G Teramo A Vicenzetto C Gasparini VR Semenzato G. T Cell Large Granular Lymphocyte Leukemia and Chronic Nk Lymphocytosis. Best Pract Res Clin Haematol (2019) 32(3):207–16. doi: 10.1016/j.beha.2019.06.006 31585621

[10] Garcia-Suarez J Prieto A Reyes E Arribalzaga K Perez-Machado MA Lopez-Rubio M. Persistent Lymphocytosis of Natural Killer Cells in Autoimmune Thrombocytopenic Purpura (Atp) Patients After Splenectomy. Br J Haematol (1995) 89(3):653–5. doi: 10.1111/j.1365-2141.1995.tb08382.x 7734372

[11] Liu S Rice L Ewton A. Reactive Nk Cell Lymphocytosis With Atypical Immunophenotype in a Chronic Hiv-Infected Patient. Cytometry B Clin Cytom (2021) 100(2):240–2. doi: 10.1002/cyto.b.21969 33211408

[12] Chan A Hong DL Atzberger A Kollnberger S Filer AD Buckley CD. Cd56bright Human Nk Cells Differentiate Into Cd56dim Cells: Role of Contact With Peripheral Fibroblasts. J Immunol (2007) 179(1):89–94. doi: 10.4049/jimmunol.179.1.89 17579025

[13] Cooper MA Fehniger TA Caligiuri MA. The Biology of Human Natural Killer-Cell Subsets. Trends Immunol (2001) 22(11):633–40. doi: 10.1016/s1471-4906(01)02060-9 11698225

[14] Juelke K Killig M Luetke-Eversloh M Parente E Gruen J Morandi B. Cd62l Expression Identifies a Unique Subset of Polyfunctional Cd56dim Nk Cells. Blood (2010) 116(8):1299–307. doi: 10.1182/blood-2009-11-253286 20505160

[15] Warren HS Skipsey LJ. Loss of Activation-Induced Cd45ro With Maintenance of Cd45ra Expression During Prolonged Culture of T Cells and Nk Cells. Immunology (1991) 74(1):78–85. 1834549 PMC1384675

[16] Morice WG. The Immunophenotypic Attributes of Nk Cells and Nk-Cell Lineage Lymphoproliferative Disorders. Am J Clin Pathol (2007) 127(6):881–6. doi: 10.1309/q49crj030l22mhlf 17509985

[17] Lima M Almeida J dos Anjos Teixeira M Queirós ML Justiça B Orfão A. The "Ex Vivo" Patterns of Cd2/Cd7, Cd57/Cd11c, Cd38/Cd11b, Cd45ra/Cd45ro, and Cd11a/Hla-Dr Expression Identify Acute/Early and Chronic/Late Nk-Cell Activation States. Blood Cells Mol Dis (2002) 28(2):181–90. doi: 10.1006/bcmd.2002.0506 12064914

[18] Rong Q Gao Y Cai Q Wang X Bai B Ping L. High Il-6 Expression in the Tumor Microenvironment Is Associated With Poor Prognosis of Patients With Extranodal Natural / Killer T-Cell Lymphoma (Enktl). Expert Rev Anticancer Ther (2021) 21(1):121–7. doi: 10.1080/14737140.2021.1853531 33397158

[19] Lam ST Huang H Fang X Wang Z Hong H Ren Q. A New Immunological Prognostic Model Based on Immunohistochemistry for Extranodal Natural Killer/T-Cell Lymphoma Patients After Non-Anthracycline-Based Chemotherapy. Cancer Manag Res (2020) 12:1981–90. doi: 10.2147/cmar.S244176 PMC7085340 32231439

[20] True LD. Quality Control in Molecular Immunohistochemistry. Histochem Cell Biol (2008) 130(3):473–80. doi: 10.1007/s00418-008-0481-0 PMC2522330 18648842

[21] Tripodi SA Rocca BJ Hako L Barbagli L Bartolommei S Ambrosio MR. Quality Control by Tissue Microarray in Immunohistochemistry. J Clin Pathol (2012) 65(7):635–7. doi: 10.1136/jclinpath-2011-200551 22461649

[22] de Mel S Li JB Abid MB Tang T Tay HM Ting WC. The Utility of Flow Cytometry in Differentiating Nk/T Cell Lymphoma From Indolent and Reactive Nk Cell Proliferations. Cytometry B Clin Cytom (2018) 94(1):159–68. doi: 10.1002/cyto.b.21529 28431200

[23] Dębska-Zielkowska J Moszkowska G Zieliński M Zielińska H Dukat-Mazurek A Trzonkowski P. Kir Receptors as Key Regulators of Nk Cells Activity in Health and Disease. Cells (2021) 10(7):4–7. doi: 10.3390/cells10071777 PMC8303609 34359951

[24] Maiers M Mehr R Raghavan M Kaufman J Louzoun Y. Editorial: Hla and Kir Diversity and Polymorphisms: Emerging Concepts. Front Immunol (2021) 12:701398. doi: 10.3389/fimmu.2021.701398 34079561 PMC8166334

[25] Pastoret C Desmots F Drillet G Le Gallou S Boulland ML Thannberger A. Linking the Kir Phenotype With Stat3 and Tet2 Mutations to Identify Chronic Lymphoproliferative Disorders of Nk Cells. Blood (2021) 137(23):3237–50. doi: 10.1182/blood.2020006721 PMC8351897 33512451

[26] Muñoz-García N Jara-Acevedo M Caldas C Bárcena P López A Puig N. Stat3 and Stat5b Mutations in T/Nk-Cell Chronic Lymphoproliferative Disorders of Large Granular Lymphocytes (Lgl): Association With Disease Features. Cancers (Basel) (2020) 12(12):14–5. doi: 10.3390/cancers12123508 PMC7760806 33255665

[27] Jerez A Clemente MJ Makishima H Koskela H Leblanc F Peng Ng K. Stat3 Mutations Unify the Pathogenesis of Chronic Lymphoproliferative Disorders of Nk Cells and T-Cell Large Granular Lymphocyte Leukemia. Blood (2012) 120(15):3048–57. doi: 10.1182/blood-2012-06-435297 PMC3471515 22859607

[28] Gasparini VR Binatti A Coppe A Teramo A Vicenzetto C Calabretto G. A High Definition Picture of Somatic Mutations in Chronic Lymphoproliferative Disorder of Natural Killer Cells. Blood Cancer J (2020) 10(4):42. doi: 10.1038/s41408-020-0309-2 32321919 PMC7176632

[29] Barilà G Teramo A Calabretto G Ercolin C Boscaro E Trimarco V. Dominant Cytotoxic Nk Cell Subset Within Clpd-Nk Patients Identifies a More Aggressive Nk Cell Proliferation. Blood Cancer J (2018) 8(6):51. doi: 10.1038/s41408-018-0088-1 29891951 PMC6002482

[30] Ha JY Cho H Sung H Jung AR Lee YS Lee SW. Superiority of Epstein-Barr Virus DNA in the Plasma Over Whole Blood for Prognostication of Extranodal Nk/T Cell Lymphoma. Front Oncol (2020) 10:594692. doi: 10.3389/fonc.2020.594692 33330083 PMC7734249

[31] Trusler O Huang Z Goodwin J Laslett AL. Cell Surface Markers for the Identification and Study of Human Naive Pluripotent Stem Cells. Stem Cell Res (2018) 26:36–43. doi: 10.1016/j.scr.2017.11.017 29227830

[32] Wei S Cao D Liu Z Li J Wu H Gong J. Dysfunctional Immunoregulation in Human Liver Allograft Rejection Associated With Compromised Galectin-1/Cd7 Pathway Function. Cell Death Dis (2018) 9(3):293. doi: 10.1038/s41419-017-0220-3 29463785 PMC5833641

[33] Newick K O'Brien S Moon E Albelda SM. Car T Cell Therapy for Solid Tumors. Annu Rev Med (2017) 68:139–52. doi: 10.1146/annurev-med-062315-120245 27860544

[34] Gomes-Silva D Srinivasan M Sharma S Lee CM Wagner DL Davis TH. Cd7-Edited T Cells Expressing a Cd7-Specific Car for the Therapy of T-Cell Malignancies. Blood (2017) 130(3):285–96. doi: 10.1182/blood-2017-01-761320 PMC5520470 28539325

[35] Gomes-Silva D Atilla E Atilla PA Mo F Tashiro H Srinivasan M. Cd7 Car T Cells for the Therapy of Acute Myeloid Leukemia. Mol Ther (2019) 27(1):272–80. doi: 10.1016/j.ymthe.2018.10.001 PMC6318703 30391141

[36] Tang J Li J Zhu X Yu Y Chen D Yuan L. Novel Cd7-Specific Nanobody-Based Immunotoxins Potently Enhanced Apoptosis of Cd7-Positive Malignant Cells. Oncotarget (2016) 7(23):34070–83. doi: 10.18632/oncotarget.8710 PMC5085138 27083001

[37] Kwong YL Chan TSY Tan D Kim SJ Poon LM Mow B. Pd1 Blockade With Pembrolizumab Is Highly Effective in Relapsed or Refractory Nk/T-Cell Lymphoma Failing L-Asparaginase. Blood (2017) 129(17):2437–42. doi: 10.1182/blood-2016-12-756841 28188133

[38] Doroshow DB Bhalla S Beasley MB Sholl LM Kerr KM Gnjatic S. Pd-L1 as a Biomarker of Response to Immune-Checkpoint Inhibitors. Nat Rev Clin Oncol (2021) 18(6):345–62. doi: 10.1038/s41571-021-00473-5 33580222

[39] Cho J Kim SJ Park WY Kim J Woo J Kim G. Immune Subtyping of Extranodal Nk/T-Cell Lymphoma: A New Biomarker and an Immune Shift During Disease Progression. Mod Pathol (2020) 33(4):603–15. doi: 10.1038/s41379-019-0392-8 31653980

[40] Bi XW Wang H Zhang WW Wang JH Liu WJ Xia ZJ. Pd-L1 Is Upregulated by Ebv-Driven Lmp1 Through Nf-Kb Pathway and Correlates With Poor Prognosis in Natural Killer/T-Cell Lymphoma. J Hematol Oncol (2016) 9(1):109. doi: 10.1186/s13045-016-0341-7 27737703 PMC5064887

[41] Feng Y Feng X Jing C Yu X Zheng Y Xu C. The Expression and Clinical Significance of Programmed Cell Death Receptor 1 and Its Ligand in Tumor Tissues of Patients With Extranodal Nasal Nk/T Cell Lymphoma. Sci Rep (2022) 12(1):36. doi: 10.1038/s41598-021-02515-5 34996890 PMC8742095

[42] Wang L Wang H Li PF Lu Y Xia ZJ Huang HQ. Cd38 Expression Predicts Poor Prognosis and Might Be a Potential Therapy Target in Extranodal Nk/T Cell Lymphoma, Nasal Type. Ann Hematol (2015) 94(8):1381–8. doi: 10.1007/s00277-015-2359-2 25865943

[43] Li P Jiang L Zhang X Liu J Wang H. Cd30 Expression Is a Novel Prognostic Indicator in Extranodal Natural Killer/T-Cell Lymphoma, Nasal Type. BMC Cancer (2014) 14:890. doi: 10.1186/1471-2407-14-890 25429803 PMC4258942

[44] Kim WY Nam SJ Kim S Kim TM Heo DS Kim CW. Prognostic Implications of Cd30 Expression in Extranodal Natural Killer/T-Cell Lymphoma According to Treatment Modalities. Leuk Lymphoma (2015) 56(6):1778–86. doi: 10.3109/10428194.2014.974048 25288491

[45] Horwitz S O'Connor OA Pro B Illidge T Fanale M Advani R. Brentuximab Vedotin With Chemotherapy for Cd30-Positive Peripheral T-Cell Lymphoma (Echelon-2): A Global, Double-Blind, Randomised, Phase 3 Trial. Lancet (2019) 393(10168):229–40. doi: 10.1016/s0140-6736(18)32984-2 PMC6436818 30522922

