# Supplementary figures and images for: Comprehensive Analysis and Summary of the Value of Immunophenotypes of Mature NK Cell Tumors for Differential Diagnosis, Treatment, and Prognosis
Source: Front Immunol. 2022 Jun 24;13:918487. doi: 10.3389/fimmu.2022.918487 (PMC9263723; doi:10.3389/fimmu.2022.918487)

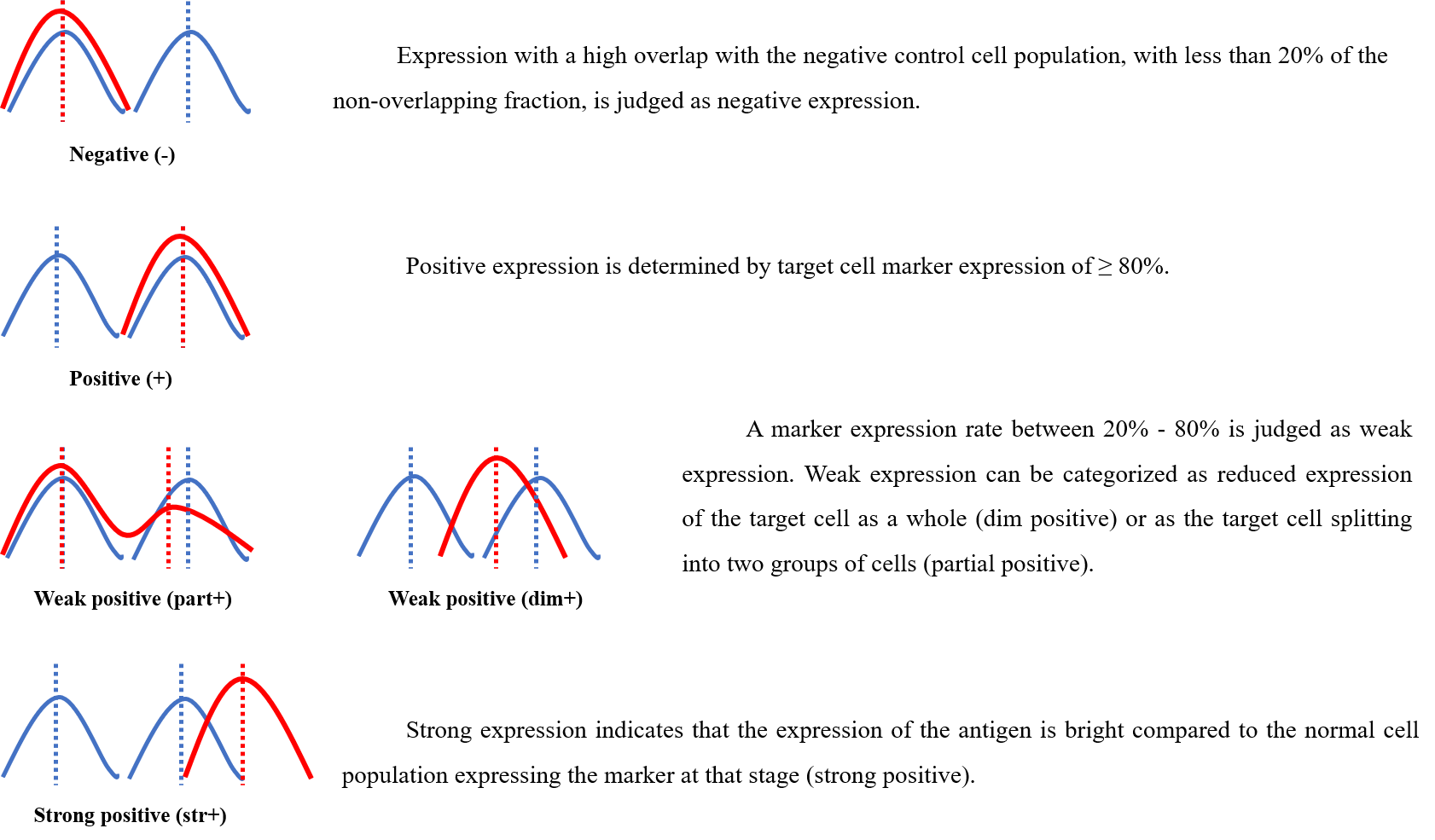

Supplement: Supplementary Figure 1 — Specific methods for defining expression intensity. According to the amount of expression of surface markers, they can be classified as non-expressed, expressed, weakly expressed, and strongly expressed. [file Image_1.tif]

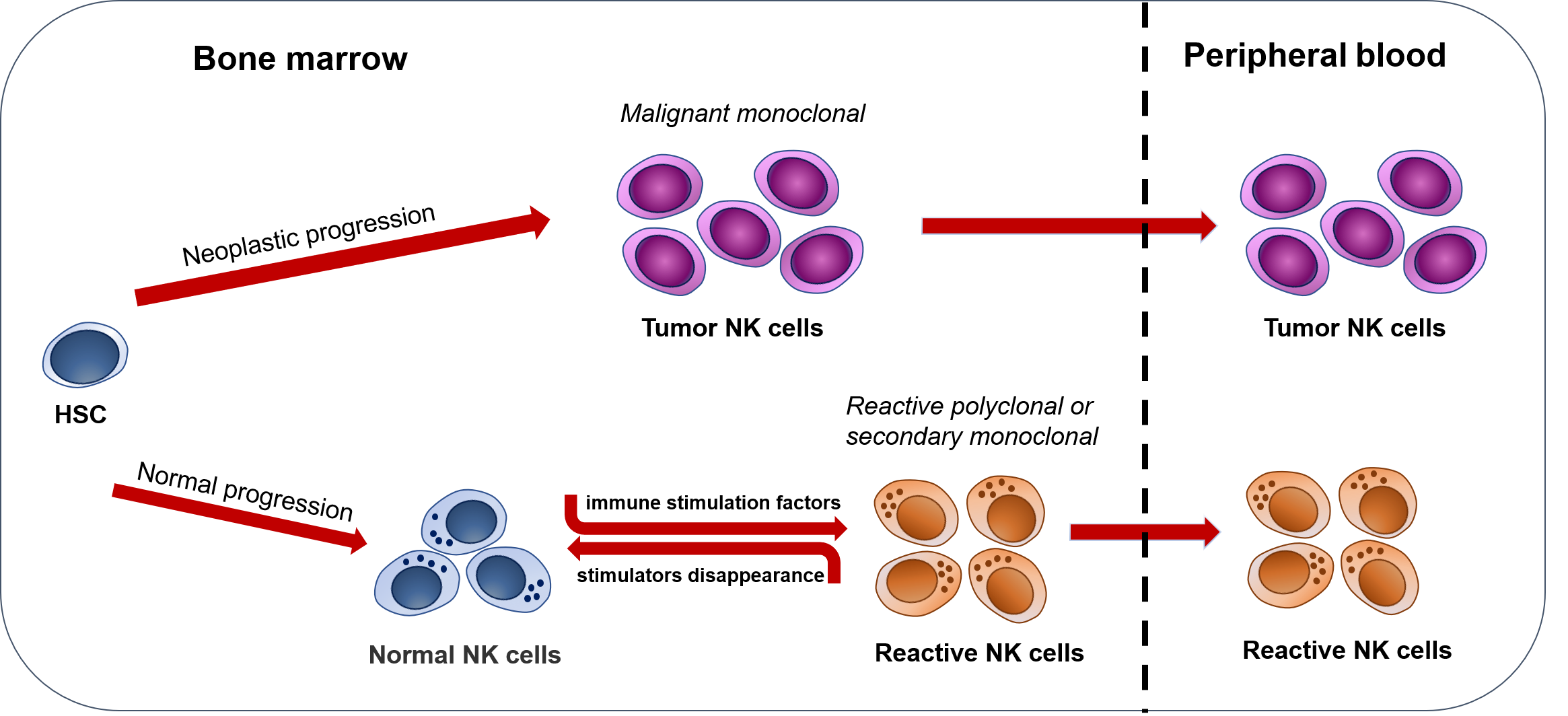

Supplement: Supplementary Figure 2 — Different progression of reactive and malignant NK cells. Reactive NK cells arise in response to immune stimulation and can induce reactive polyclonal or secondary monoclonal proliferation. Reactive NK cells tend to disappear after the disappearance of the stimulus. [file Image_2.tif]
