# Supplementary material for: Comprehensive Analysis and Summary of the Value of Immunophenotypes of Mature NK Cell Tumors for Differential Diagnosis, Treatment, and Prognosis
Source: Front Immunol. 2022 Jun 24;13:918487. doi: 10.3389/fimmu.2022.918487 (PMC9263723; doi:10.3389/fimmu.2022.918487)
Supplement: Supplementary Table 1 — The clone number of the antibodies used. [file Table_1.docx]

| Supplementary Table 1: The clone number of the antibodies used. | | | | |
| --- | --- | --- | --- | --- |
| Tubes | **Molecular target** | **Fluorescein** | **Clone** | **Manufacturers** |
| 1 | CD57 | FITC | HNK-1 | BD |
| 1 | CD8 | PE | SK1 | BD |
| 1 | CD4 | APC-cy7 | SK3 | BD |
| 1 | PD-1 | BV605 | EH12.1 | BD |
| 2 | CD3 | FITC | SK7 | BD |
| 2 | CD2 | PE | RPA-2.10 | BD |
| 2 | CD5 | PerCP | L17F12 | BD |
| 3 | Ki-67 | FITC | 35/KI-67 | BD |
| 3 | CD30 | PE | Ber-H83 | BD |
| 3 | HLA-DR | APC | TU36 | BD |
| 3 | CD38 | BV605 | HB7 | BD |
| 4 | CD158e1 | FITC | DX9 | BD |
| 4 | CD158b | PE | CH-L | BD |
| 4 | CD158i | APC | 179315 | BD |
| 4 | CD158a | BV421 | HP-3E4 | BD |
| 5 | Granzyme B | PE | GB11 | BD |
| 5 | CD94 | APC | HP-3D9 | BD |
| 5 | Perforin | BV421 | ΔG9 | BD |
| 1,2,3 | CD7 | V450 | M-T701 | BD |
| 2,4,5 | CD16 | APC-cy7 | 3G8 | BD |
| 1,3,4,5 | CD3 | PerCP | SK7 | BD |
| 1,2,3,4,5 | CD56 | PE-cy7 | B159 | BD |
| 1,2,3,4,5 | CD45 | V500 | HI30 | BD |
