# Supplementary material for: Comprehensive Analysis and Summary of the Value of Immunophenotypes of Mature NK Cell Tumors for Differential Diagnosis, Treatment, and Prognosis
Source: Front Immunol. 2022 Jun 24;13:918487. doi: 10.3389/fimmu.2022.918487 (PMC9263723; doi:10.3389/fimmu.2022.918487)
Supplement: Supplementary Table 2 — p-values for differences between groups. [file Table_2.docx]

| **Supplementary Table 2:** **p-values for differences between groups.** | | | | | | | | | |  |
| --- | --- | --- | --- | --- | --- | --- | --- | --- | --- | --- |
|  | p-value① | | | p-value② | | | p-value③ | | | p-value④ |
|  | ANKL  v.s.  ENKTL | ANKL  v.s.  NK-CLPD | ENKTL  v.s.  NK-CLPD | ANKL  v.s.  RNKL | ENKTL  v.s.  RNKL | NK-CLPD  v.s  RNKL | ANKL  v.s.  NNK | ENKTLv.s.  NNK | NK-CLPD  v.s.  NNK | RNKL  v.s.  NNK |
| Age, years, mean (SD) | 0.362 | 0.003* | 0.002* | 0.035* | 0.021* | 0.679 | 0.049* | 0.046* | 0.350 | 0.596 |
| Male sex | 0.470 | 1.000 | 0.381 | 0.680 | 0.114 | 1.000 | 0.487 | 0.033* | 0.691 | 1.000 |
| **Symptoms at onset of illness** | | | | | | | | | | |
| Fever | 0.031* | 0.036* | 1.000 | <0.001* | 0.057 | 0.342 | <0.001* | <0.001* | 0.003* | 0.095 |
| Fatigue | 0.006* | 0.309 | 0.228 | 0.010* | 0.062 | 0.342 | <0.001* | 0.526 | 0.091 | 1.000 |
| Multiple lymph nodes are swollen throughout the body | 0.493 | 0.380 | 0.097 | 0.019* | <0.001* | 0.183 | 0.001* | <0.001* | 0.074 | NS |
| Hepatomegaly | 0.002* | 0.040* | 1.000 | 0.006* | 1.000 | 0.444 | <0.001* | 0.491 | 0.286 | NS |
| Splenomegaly | 0.683 | 0.137 | 0.381 | 0.003* | 0.007* | 0.321 | <0.001* | <0.001* | 0.142 | 1.000 |
| **Complications** | | | | | | | | | | |
| Hemophagocytic syndrome | 0.007* | 0.008* | 0.676 | 0.001* | 0.007* | 0.321 | 0.002* | 0.074 | 0.003* | 0.333 |
| Hyperlipidemia | 0.491 | 0.386 | 1.000 | 0.002* | 0.021* | 0.118 | <0.001* | 0.002* | 0.038* | 1.000 |
| Hypoproteinemia | 0.305 | 0.074 | 0.379 | 0.006* | 0.071 | 0.444 | 0.023* | 0.175 | 1.000 | 0.532 |
| Hepatic dysfunction | 0.275 | 0.010* | 0.053 | <0.001* | <0.001* | 0.118 | <0.001* | <0.001* | 0.038* | 1.000 |
| Renal dysfunction | 0.015* | 0.183 | 1.000 | 0.002* | 0.291 | 0.183 | 0.002* | 0.420 | 0.555 | 0.540 |
| Coagulation dysfunction | 0.123 | 0.002* | 0.043* | <0.001* | 0.020* | 0.608 | <0.001 | 0.006* | 0.651 | 1.000 |
| Multiple organ failure | 0.146 | 0.022* | 0.291 | 0.019* | 0.291 | NS | 0.001* | 0.051 | NS | NS |
| **Laboratory results** | | | | | | | | | | |
| White blood cell <4 × 10^9^/L | 0.493 | 0.022* | 0.002* | 0.421 | 0.126 | 1.000 | 0.487 | 0.069 | 1.000 | 1.000 |
| Neutrophil <2 × 10^9^/L | 1.000 | 0.662 | 0.405 | 0.653 | 0.686 | 0.321 | 0.704 | 0.745 | 0.371 | 1.000 |
| Red blood cells<4 × 10^9^/L | 0.276 | 0.002* | 0.027* | <0.001* | 0.001* | 0.608 | 0.031* | 0.473 | 0.200 | 0.019* |
| Hemoglobin<105 g/L | 0.027* | 0.039* | 1.000 | 0.002* | 0.259 | 0.630 | 0.024* | 1.000 | 1.000 | 0.260 |
| Platelets<100 × 10^9^/L | 1.000 | 0.081 | 0.074 | 0.035* | 0.016* | 1.000 | 0.704 | 1.000 | 0.200 | 0.056 |
| RDW> 15% | 0.710 | 0.624 | 1.000 | 0.011* | 0.020* | 0.145 | 0.467 | 1.000 | 1.000 | 0.050 |
| Serum LDH > 250 U/L | 0.683 | 0.008* | 0.032* | 0.032* | 0.114 | 0.638 | <0.001* | 0.001* | 1.000 | 0.384 |
| Serum ALP > 100 U/L | 0.150 | 0.052 | 0.412 | 0.003* | 0.057 | 0.608 | 0.001* | 0.031* | 0.651 | 1.000 |
| Serum β2-MG > 3 mg/L | 0.710 | 0.081 | 0.206 | 0.035* | 0.057 | 1.000 | <0.001* | <0.001* | 0.058 | 0.095 |
| Serum ferritin > 150 ng/mL | 0.522 | <0.001* | <0.001* | <0.001* | <0.001* | 1.000 | <0.001* | <0.001* | 1.000 | 0.584 |
| Serum EBV-DNA >5.0 × 10^3^ copies/mL | 0.112 | 0.011* | 0.228 | <0.001* | 0.007* | 0.275 | <0.001* | <0.001* | 0.017* | 0.333 |
| ①Differences between two of the three NK cell tumors; ②Differences between each of the three NK cell tumors and RNKL; ③Differences between each of the three NK cell tumors and NNK; ④The difference between RNKL and NNK.  The algorithm of the P-value between the two groups for the age parameter used the T test; The P-value of the other parameters used the Fisher’ exact probabilities.  ***,** P-value less than or equal to 0.05 was considered statistically significant. | | | | | | | | | | |
